# Forecasting Chikungunya spread in the Americas *via* data-driven empirical approaches

**DOI:** 10.1186/s13071-016-1403-y

**Published:** 2016-02-29

**Authors:** Luis E. Escobar, Huijie Qiao, A. Townsend Peterson

**Affiliations:** Veterinary Population Medicine, College of Veterinary Medicine, University of Minnesota, St. Paul, MN USA; Minnesota Aquatic Invasive Species Research Center, University of Minnesota, St. Paul, MN USA; Center for Global Health and Translational Science, SUNY Upstate Medical University, Syracuse, NY USA; Key Laboratory of Animal Ecology and Conservation Biology, Institute of Zoology, Chinese Academy of Sciences, Beijing, China; Biodiversity Institute, University of Kansas, Lawrence, KS USA

**Keywords:** Epidemic, Transmission, Disease model, Vector-borne, Passenger flow

## Abstract

**Background:**

Chikungunya virus (CHIKV) is endemic to Africa and Asia, but the Asian genotype invaded the Americas in 2013. The fast increase of human infections in the American epidemic emphasized the urgency of developing detailed predictions of case numbers and the potential geographic spread of this disease.

**Methods:**

We developed a simple model incorporating cases generated locally and cases imported from other countries, and forecasted transmission hotspots at the level of countries and at finer scales, in terms of ecological features.

**Results:**

By late January 2015, >1.2 M CHIKV cases were reported from the Americas, with country-level prevalences between nil and more than 20 %. In the early stages of the epidemic, exponential growth in case numbers was common; later, however, poor and uneven reporting became more common, in a phenomenon we term "*surveillance fatigue*." Economic activity of countries was not associated with prevalence, but diverse social factors may be linked to surveillance effort and reporting.

**Conclusions:**

Our model predictions were initially quite inaccurate, but improved markedly as more data accumulated within the Americas. The data-driven methodology explored in this study provides an opportunity to generate descriptive and predictive information on spread of emerging diseases in the short-term under simple models based on open-access tools and data that can inform early-warning systems and public health intelligence.

**Electronic supplementary material:**

The online version of this article (doi:10.1186/s13071-016-1403-y) contains supplementary material, which is available to authorized users.

## Background

**Fig. 1:**
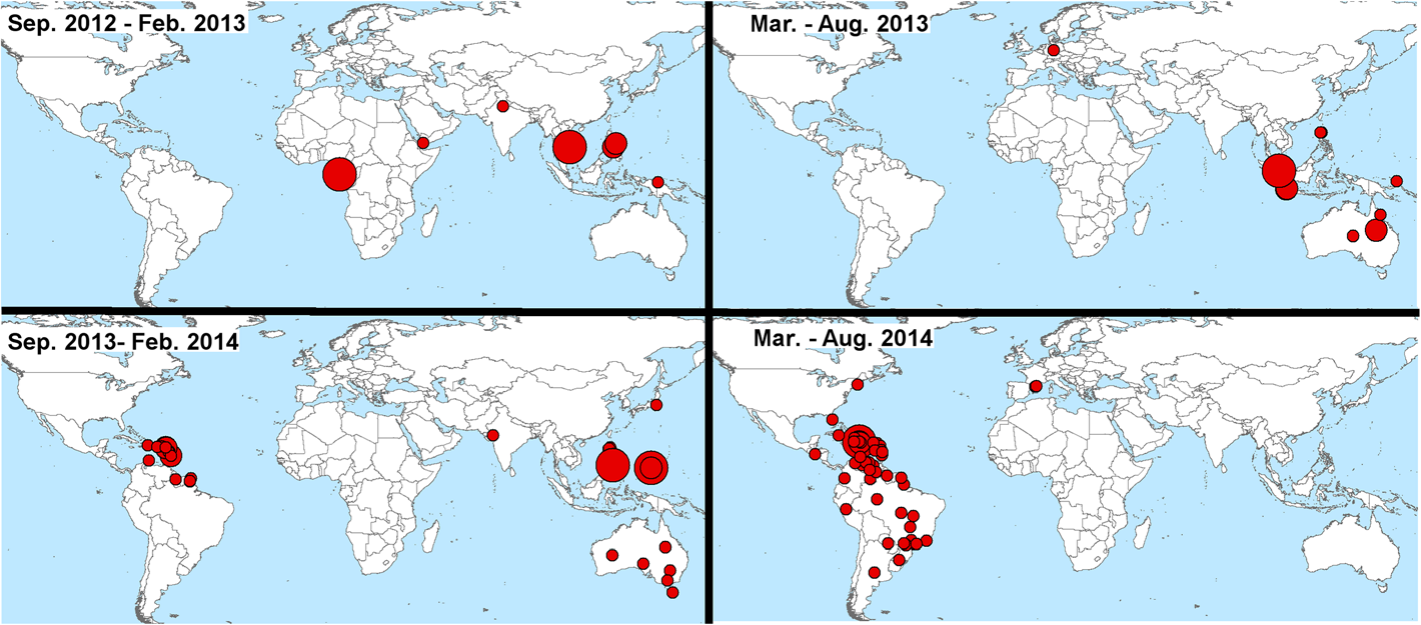
Geographic distribution of Chikungunya case concentrations worldwide. Snapshots of Chikungunya distribution between September 2012 and August 2014, according to ProMed data from [68]

Chikungunya virus (CHIKV; genus *Alphavirus*) is endemic to Africa and Asia. It comprises three genotypes (East-Central-South African, West African, Asian); the Asian genotype invaded the Americas in 2013, quickly developing autochthonous transmission [1, 2] (Fig. 1). CHIKV is transmitted by several mosquito species, but *Aedes albopictus* and *A. aegypti* are the principal vectors, and have proven highly competent for CHIKV transmission across the Americas [3]. These vector species have broad potential geographic distributions across the Americas under current and future climate conditions [4], such that the virus sees enormous opportunities for spread.

In the early stages of the spread of this disease in the Americas (Fig. 1), the spatial structure of CHIKV occurrences in the Caribbean was explicable in terms of distances between countries [5]. However, considering the broad current extent of the epidemic, a more detailed biogeographic and ecological approach may be needed to identify and anticipate current and future trends in the CHIKV epidemic. However, the data necessary for correlative ecological niche models at coarse scales are still highly biased spatially (e.g., collected along roadsides) [6, 7], such that comprehensive risk maps are probably not feasibly developed by those methods solely. With more than a million cases to date in the Americas, Cauchemez et al. [5] found that CHIKV models based on the first 1–3 months of data changed considerably in terms of predicted incidence as more data became available. Furthermore, differences in quality of CHIKV reporting among countries across the Americas suggest that some countries report individual cases in detail, whereas others accumulate hundreds of cases before reporting begins [5]. Finally, because more than 25 % of CHIKV-infected individuals may be asymptomatic [8], and CHIKV symptoms may be confused with dengue fever [9], reporting can be incomplete or irregular, further complicating modeling efforts.

The CHIKV epidemic in the Americas represents an impressive case of an emerging infectious disease at continental scales that demands detailed understanding and prediction of its spread (Fig. 1). This epidemic provides an opportunity to explore the utility and importance of novel computational tools and data streams in disease risk mapping during epidemics. We aimed to explore a data-driven, ecological approach to forecast CHIKV spread across the Americas. In this paper, to assess model performance from weekly CHIKV reports, we integrate air travel information, geographic distance and connectivity, and climatic suitability for vector species to understand and anticipate the spread of CHIKV in the Americas. Considering that a simple approach is appropriated when there is no detailed knowledge of an infectious disease, our CHIKV model is developed based on few parameters to minimize the need for assumptions; additionally, our model is based on open-access data and tools, which may permit further implementation of our methodology as an alternative in exploring infectious diseases systems affecting broad geographic areas and lacking in the understanding of the basic biology necessary for models requiring complex parameterization.

## Methods

### Exploring patterns of surveillance

#### Overall case numbers

With large numbers of cases diagnosed in many countries, and so many exported cases [10], probabilities of dispersal and establishment are relatively high. Thus, a deterministic approach was used in this exploration. Hence, we explored CHIKV case numbers in each country in one-week time steps, considering the sum of pre-existing CHIKV cases at the time of prediction, number of cases imported, and number of cases generated locally by autochthonous transmission. We integrated these components in Eq. (1) as follows:

1 $$ N{T}_{i,j} = N{T}_{i-1,j} + N{I}_{i,j} + N{L}_{i,j} $$

where *NT*_*i,j*_ is the cumulative number of individuals infected by week *i* in country *j*, as reported by the Pan American Health Organization (PAHO; [11]); simulating a scenario of an ongoing epidemic requiring immediate predictions our model estimates started in week 35 of the outbreak in the Americas (i.e., August 2014 represents *i = 0*). *NI*_*i,j*,_ is the number of cases imported in week *i* into country *j*. Finally, *NL*_*i,j*_ is the number of cases generated within country *j* in week *i* by local transmission.

#### Imported cases

Imported cases were based on a population-growth-via-immigration approach. Cases were estimated from a detailed evaluation of connectivity among cities of the Americas *via* air travel. At this early stage of the outbreak, and given the insular nature of the initial suite of countries infected, we were comfortable in neglecting ship and ground travel, which may not be tenable in later stages of the outbreak. Our air travel connectivity model was based roughly on Brockmann and Helbing [12]. However, the principal data source for Brockmann and Helbing [12] was the Official Airline Guide, Ltd. [13], which is restrictive to the scientific community as it targets the travel industry as customers and clients, and is enormously expensive. To overcome this obstacle, *via* correlative approaches, we derived a data set that correlates closely with the closed-source industry data, but that was derived from openly available sources, appears representative of numbers of passengers on flights, and is free of cost, as follows.

We estimated passenger flow *via* a correlative model relating airport and route characteristics to passenger data, all information that could be obtained openly. Specifically, we collected air travel route characteristic data from the OurAirports data repository [14]. We focused on 65,247 air travel routes, and assembled information including origin, destination, flight distance, aircraft type, and number of seats by aircraft. We mapped 2,632 airports using their longitude, latitude, number of runways, and runway area, for a total of 364 locations and 39,376 runways, at the level of city or province in 114 countries globally, (Additional file 1). We estimated passenger flow by associating flight flow and aircraft-specific passenger capacity. Data regarding aircraft characteristics (i.e., numbers of seats) were drawn from Wikipedia [15]. Once aircraft routes and passenger data were collected, collated, and formatted, we developed a random forests model to relate route, airport, region, and runway data to passenger flow in the United States-connected flight dataset as follows (see details in Additional file 1). We validated the travel connectivity model by (i) comparing model predictions with detailed numbers of passengers per month on routes city-to-city across the United States (U.S.) using more limited traffic data provided by the U.S. Department of Transportation ([16]; see Additional file 1) and (ii) worldwide using an independent data set (i.e., the top 10 routes in the world, sourced from [17]). We assumed that sources of CHIKV for further spread in the Americas would be the regions of the Americas already infected, and thus neglected the possibility of additional introductions from Europe, Asia, or Africa.

*NI*_*i,j*_ was estimated in Eq. (2) based on passenger flow from all 51 countries in the Americas with non zero ongoing local transmission as

2 $$ N{I}_{i,j}=k{\displaystyle {\sum}_{x=1}^{51}{p}_{i,j}\kern0.5em {t}_{i,j}} $$

where *p*_*i,j*_ is the prevalence of CHIKV infection at time *i* from country *j*. This prevalence was calculated in Eq. (3) as:

3 $$ {p}_{i,j}=\frac{N{T}_{i-1}}{y} $$

where *NT*_*i*-1_ is the number of cases and y represents the total population of the country (source: [18]). The second part of equation 2, (t_*i*,*j*_), represents human movements (by air, in this case) from the infected country *j* to other countries at time *i*, derived from our passenger traffic flow calculations (Additional file 1). Finally, as an element of equation 2, we derived *k*, a scalar value derived empirically, based on the assumption that case occurrences in the United States will have been detected and reported rather comprehensively. Specifically, we compared our raw estimates of numbers of passengers coming into the United States (where reporting appears to have been constant and more or less complete) over weeks 30–35 against numbers of imported cases reported in the United States, which corresponds roughly to an estimate of “ease of infection” from travelers. We calculated the proportion of incoming travelers that translated into reported infections as *k* = 0.001269, and used this parameter value to correct imported case estimates for all countries.

#### Local transmission

*NL*_*i,j*_ in equation 1 was approximated using a simple data-driven, population-growth approach for countries with ongoing local transmission, based on patterns of accumulation of case reports in the PAHO dataset. To estimate local CHIKV transmission rates for each country *j*, we fitted a diverse family of curves to reported numbers of human cases, and assessed each for fit in terms of proportion of variance explained. Population-growth response shapes were chosen according to the diverse trajectories of increase of numbers of human cases in each country. We assumed that each model would take into account the reporting biases of its country. Models included linear as Eq. (4), logarithmic Eq. (5), exponential Eq. (6), and polynomial Eq. (7) regressions as

4 $$ N{L}_i{,}_j=a+ bi $$

5 $$ N{L}_i{,}_j=a+b \ln i $$

6 $$ N{L}_i{,}_j=a{b}^i $$

7 $$ N{L}_i{,}_j=a+{b}_1i+{b}_2{i}^2 $$

were *i* is the week of forecast in country *j*, *a* is the intercept, *b* is the constant slope of the line, and *ln* is the natural logarithm.

Predictions were evaluated *via* comparisons with real PAHO reports for each country. After an initial prediction (i.e., August or week 35 of the epidemic), models were re-calibrated by adding cases generated in the following weeks from August 2014 to January 2015. We assessed model performance *via* estimating the percent of *NT* deviation of model predictions from actual *NT*_*i,j*_ values according to PAHO reports. Failure rate was determined by comparing predictions against number of cases reported by February 2015, calculated in Eq. (8) as

8 $$ {F}_{i,j}=100\left(\frac{\widehat{N}{T}_{i,j}-N{T}_{i,j}}{N{T}_{i,j}}\right) $$

where *F*_*i,j*_ is the percent failure for predictions in week *i* in country *j*. $\widehat{N}{T}_{i,j}$ is the number of CHIKV cases predicted by models in week *i* and *NT*_*i,j*_ is the number of cases observed according to PAHO reports by February 2015.

### Transmission hotspots

To explore CHIKV potential in the Americas further and in detail finer than the country level, we focused on the ecology of the vectors and developed ecological niche models (ENM) for the two relevant mosquito species. Thus, we attempted to estimate the environmental conditions where mosquitoes occur, as an approximation of the fundamental niche [19]. To provide biological interpretation to model outputs, we assumed that fundamental niches should have a multidimensional ellipsoid form as described previously theoretically and empirically [20–25]. We also assumed that transmission is limited at least in broad terms by climatic considerations [26, 27]. CHIKV basic reproductive number *R*_0_ tends to be highest at around 25 °C temperature and 200 mm precipitation [27], which were central values of the climate conditions studied. Thus, we assumed that ideal conditions for high CHIKV transmission would be found at central values of suitable conditions identified in the ENM for the vectors. We further assumed that transmission of the virus depends on its vectors, in terms of their activity, abundance, and dispersal capability [26]. We used this knowledge to explore the most suitable areas at global scales in terms of niche centrality, as a proxy of high *R*_0_ of mosquito populations [28], and then extracted such information for the Americas. These niche centrality ideas have seen considerable exploration and testing in previous studies [20–22, 28, 29], and suggest that spatial variation of vector abundance can be explained by niche requirements [23, 24]. Thus, ecological niches were estimated using a climate envelope, based on a minimum-volume ellipsoid describing ecological features of vector occurrence based on the environmental range occupied by the species [24, 30, 31], instead of the classic correlative ENM methods of difficult biological interpretation [19]. Our approach is described in the paragraphs that follow.

Geographic coordinates of focal species of mosquitoes were used to calibrate ENMs to characterize climate conditions within which they are able to establish and maintain populations [32]. As we aimed to establish a best proxy of the species’ fundamental niche from which to estimate its centroid, we used vector occurrence data across the entire geographic distributions of the species [29]. Primary occurrence data (i.e., data documenting occurrences of individual animals at points in time and space) for *Aedes aegypti* and *A. albopictus* were drawn from Campbell et al. [4], who in turn had obtained them from 4 open-access data sources: VectorMap [33], Atlas of Living Australia [34], *species*Link [35], and the Global Biodiversity Information Facility [36]. Data for the two focal species (2,108 and 8,040 records, respectively) were used to calibrate ENMs. We characterized mosquito responses to climate patterns over recent decades (i.e., 1950–2000) *via* the WorldClim climate archive [37]. We used climate data at ~4 km spatial resolution, specifically annual mean temperature, mean diurnal temperature range, isothermality, temperature seasonality, maximum temperature of the warmest month, minimum temperature of the coldest month, temperature annual range, mean temperatures of the warmest and coldest quarters, annual precipitation, precipitation of the wettest and driest months, precipitation seasonality, and precipitation of the wettest quarter, mean temperature of the wettest and driest quarters, precipitation of the warmest and coldest quarters, and precipitation of driest quarter. We performed principal components analysis (PCA) on these climatic variables to reduce the number of and correlation among them. The first three components explained 84.9 % of the overall variance in the variables.

We estimated an ENM as a minimum-volume ellipsoid (MVE) in a multidimensional environmental space for each vector species. The environmental space was represented using the first three principal components from global climate variables [25, 38], and were used as axes by which to define the multidimensional environmental space using the freely-available ENM software NicheA [39]. Semi-axes with which to build the MVE were estimated based on Euclidean distances between mosquito occurrence points displayed in the environmental space (see details in Additional file 2). MVEs were developed using NicheA [31]. Once the ENM MVEs for the vector species were constructed, we divided each MVE into 100 layers summarizing proximity to the niche centroid (Additional file 2); these layers were then projected into geographic space to identify areas close to or far from the ENM centroid. The metric to measure the distance to the niche centroid and translate this information into a continuous geographic map was developed for this study, and is implemented in the toolbox of NicheA (version 3.0.1; http://nichea.sourceforge.net/). Finally, country average values of niche centrality distance of both vector species and CHIKV prevalences from PAHO were compared using regression analysis. We explored CHIKV potential in relation to the gross domestic product (GDP) of each country [15] in countries reporting CHIKV cases across the Americas. Statistical and spatial analysis were developed using R [40] and ArcGIS 10.2 [41].

## Results and discussion

### Patterns of surveillance effort

**Fig. 2:**
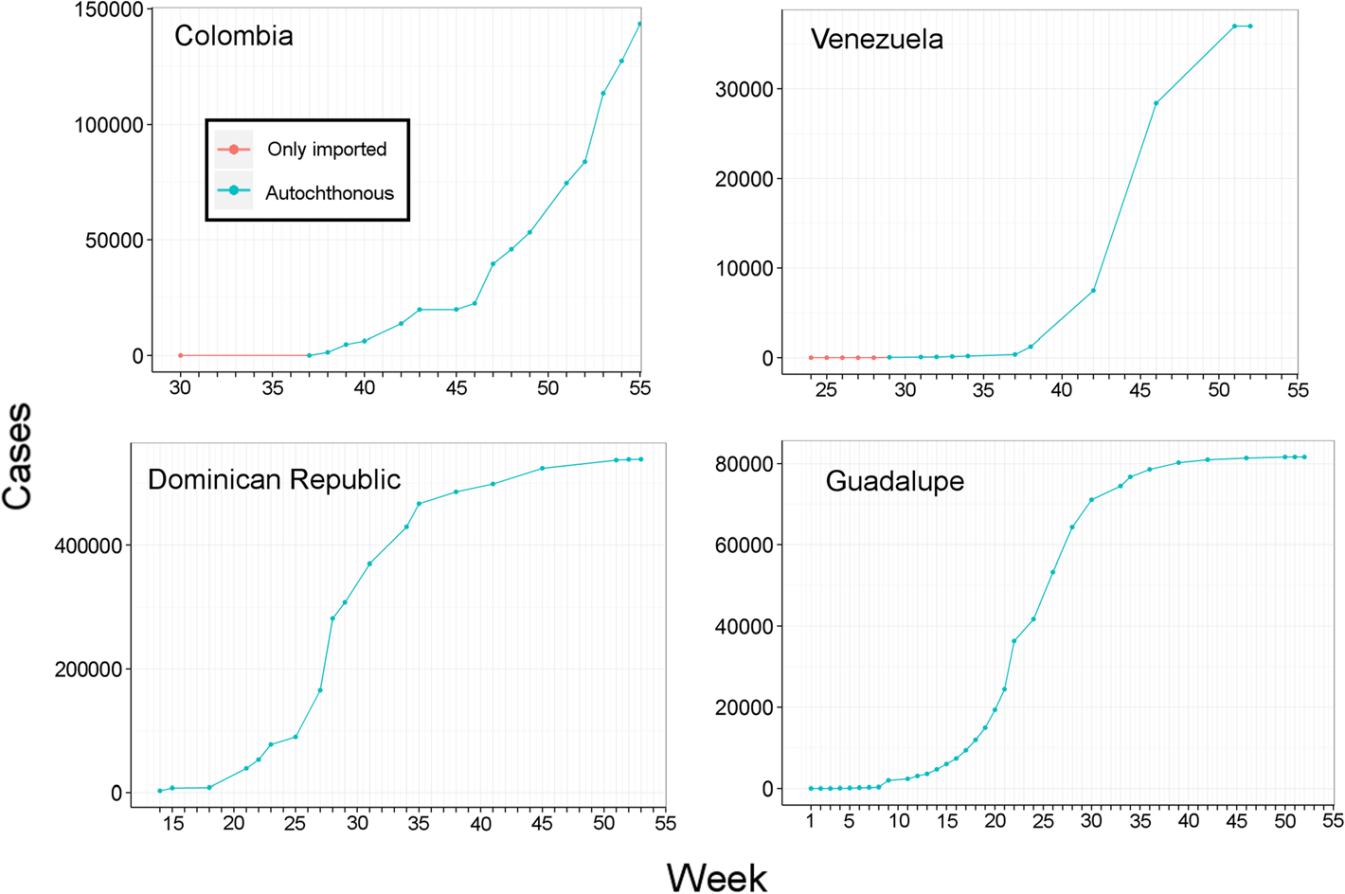
Reported Chikungunya cases. Imported cases (red line) were reported since the epidemic began in the country, followed by local transmission (blue line). Top left: Colombia showing an exponential-like shape of the line. Top right: Venezuela showing a logistic-like line and surveillance fatigue state in late weeks. Bottom: Dominican Republic (left) and Guadalupe (right) with exponential growth in early stages of the epidemic followed by logarithmic growth. The *y* axis denotes the number of accumulated cases, *x* axis denotes the week number of the epidemic according to PAHO

**Fig. 3:**
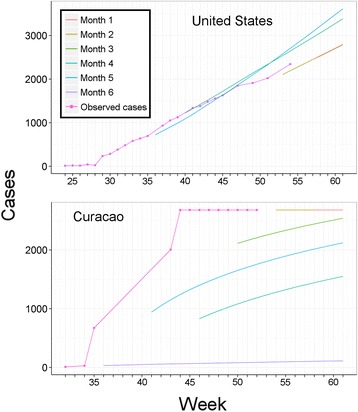
Comparison of observed versus predicted Chikungunya cases in Curacao and the United States. Incomplete, intermittent, and delay in reports generated inaccurate model calibration with consequent incorrect predictions. Top: United States. Consistent patterns of report submission allowed us to anticipate imported cases with numbers predicted close to real numbers of cases. Bottom: Curacao. While observed reports (red line) showed low increases in the first two weeks of the outbreak, the country was characterized by dramatic increases of cases with irregular reporting accumulating numbers of cases such that we could not generate correct forecasts for the following six (purple), five (blue), four (dark green), and three months (light green)

By late January 2015, 1.209,158 cases of CHIKV infection had been reported by countries in the Americas, with calculated prevalences ranging from nil (Uruguay) to 20.3 % (Martinique), and a median across countries of 0.3 %. Accumulation of case numbers in official submissions were characterized generally by exponential (e.g., Colombia; Fig. 2) or linear (e.g., United States; Fig. 3) initial growth, followed by logarithmic-like growth (e.g., Guadalupe, El Salvador; Fig. 2 and Additional file 3), with several countries ceasing reporting in recent months (e.g., Suriname, Haiti; Additional file 3). Although in many countries, cases are diagnosed and reported nationally and internationally as they occur (e.g., Colombia), other countries (e.g., Venezuela; Fig. 2) delayed in diagnosing and reporting cases; still others (e.g., Dominican Republic; Fig. 2 and Additional file 3) appeared to enter into sustained reduction of reporting, in fact, after active initial reporting and tracking, fewer cases were reported, probably not reflecting a slowdown in actual numbers of cases.

In our air passenger flow estimation, the best final model omitted month as a predictor variable, and explained 90.1 % of total overall variance in the data set (Additional file 1). From our worldwide validation, the model explained 73.0 % of variation in passenger numbers (*P* = 0.0016, *r*^2^ = 0.73), indicating considerable predictive power as regards passenger flow. We note that such correlative modeling of passenger flow represents a zero-cost, open-source segment of our methodology that could nonetheless be replaced by industry data, if the high cost were to be outbalanced by desire for less overall variance in the data.

**Fig. 4:**
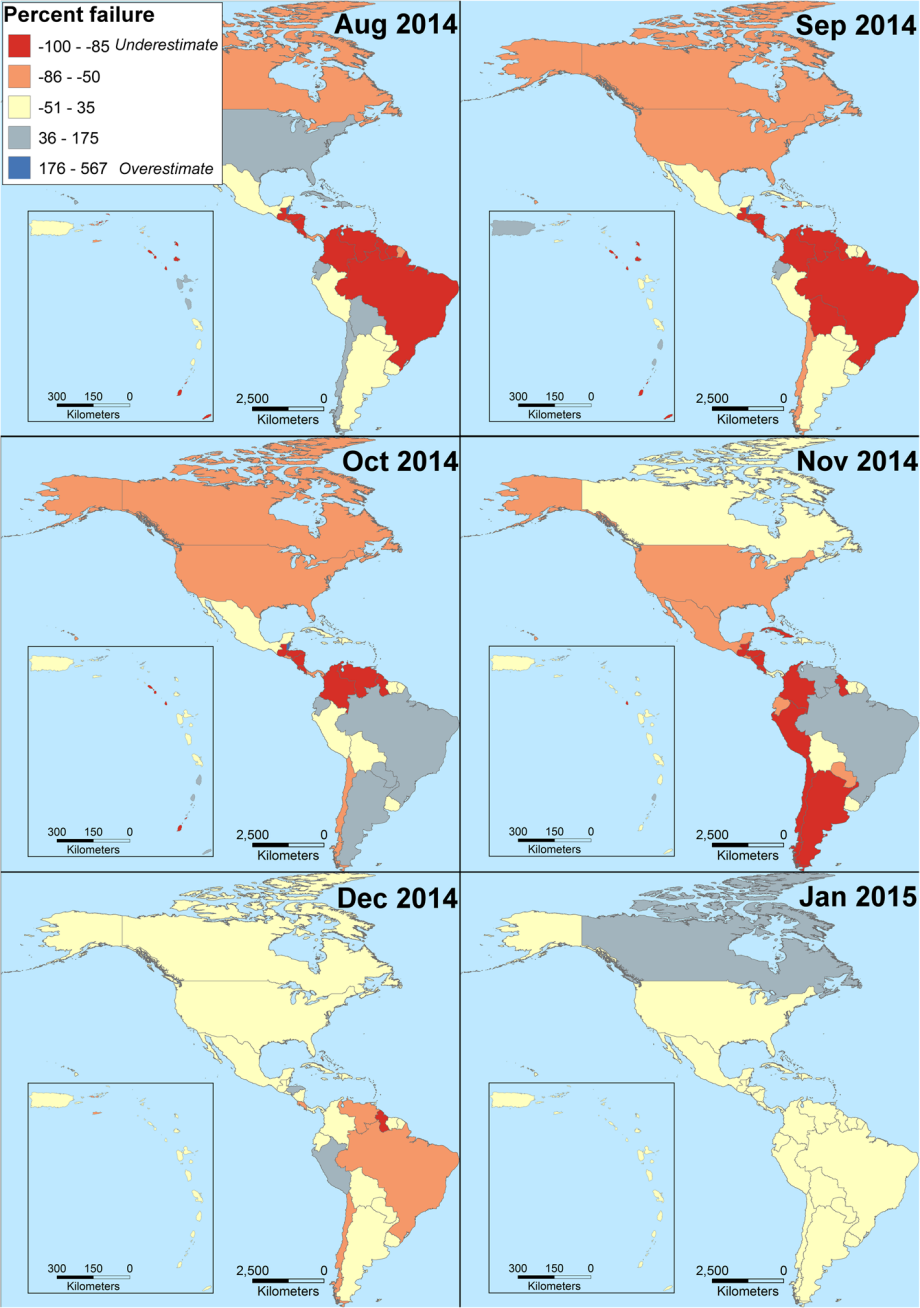
Evaluation of Chikungunya transmission model failure rates, as a function of time and associated amount of data available for model calibration. Models developed with data for August 2014, September 2014, October 2014, November 2014, December 2014, and January 2015 were compared against PAHO reports in February 2015 to assess model performance from six months to one month of anticipation respectively. Models improved in terms of fit between predicted and observed Chikungunya cases where more information was included in late models. For intervention purposes, under prediction of cases (red) was more undesirable than overprediction of cases (dark blue)

**Fig. 5:**
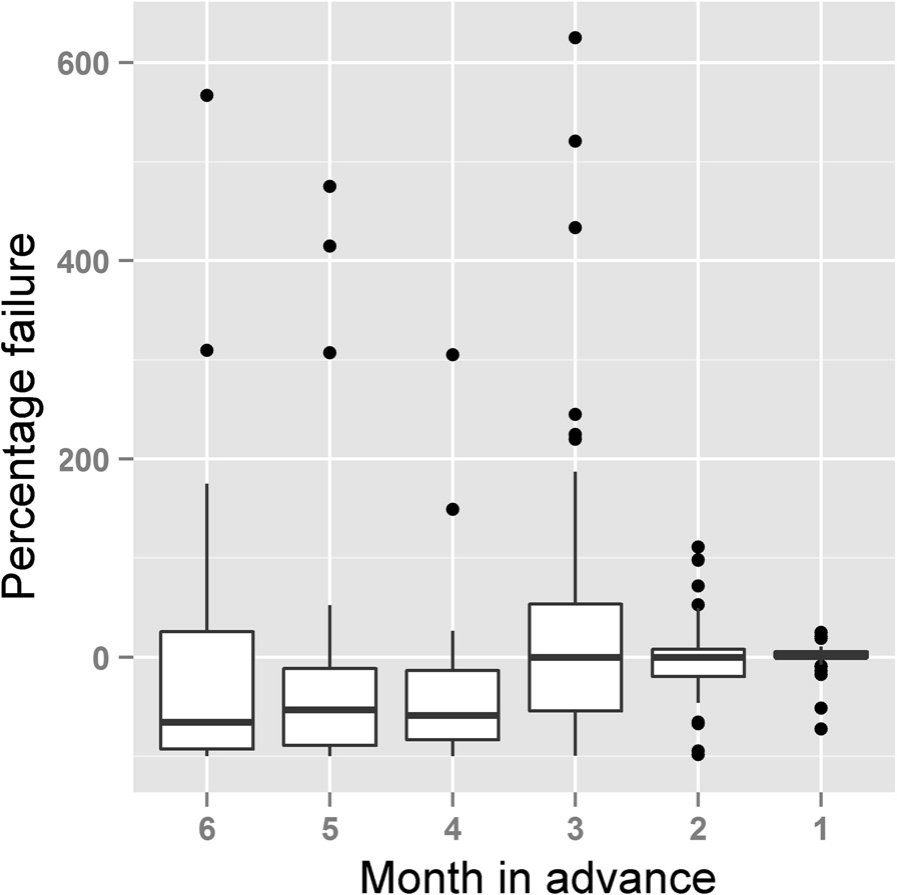
Variability during Chikungunya forecasting. Percentage failure among countries in the Americas (boxplots) was measured from predictions between August 2014 and January 2015 to assess model predictions developed from six to one month of prior PAHO reports in February 2015, on which predictions were based. The metric identify the match between real cases reported the last month of the study and models developed six or one month in advance (from left to right). Negative values represent under prediction (i.e., cases below the real report) and positive values represent overprediction (i.e., cases above the real report). Failure = 0 represents prediction matching the real number of cases reported. Notice that late models developed with more data accumulated were more close to the real reports

We inspected actual accumulation of cases in comparison to model predictions, and explored departures between the two as either model failures or biases introduced by imperfect diagnosis and reporting. Several countries showed pauses in epidemiological reporting, resulting in models that failed to anticipate future CHIKV case numbers (e.g., Curacao; Fig. 3). Using the model failure metric, when more data were added to models in final months, models tended to fail less. We assessed predictions by country for February 2015 against real reports, from models calibrated with data for August 2014, September 2014, October 2014, November 2014, December 2014, and January 2015 (Fig. 4). We found that predictions early in the epidemic contrasted dramatically in accuracy with the more informed predictions late in the epidemic that resembled the real case numbers (Fig. 5); hence, early and late predictions generated different epidemic landscapes with considerable underestimation of cases in early forecasts (Fig. 4). This result is of special concern, considering that, for public health interventions, a worst case scenario, overestimating infectious, may ensure a better response from health authorities compared to a scenario of under prediction that can be overwhelmed by real case number [42]. For example, in the Dominican Republic, CHIKV cases were underreported due to the high number of cases that overwhelmed the national diagnostic capacities (A.M. Stewart-Ibarra, *pers. comm.*). The considerable heterogeneity on the country-by-country reporting of cases directly influenced the output of the models, limiting the ability of the models to estimate the real burden of the disease at early stages of the forecast, especially from countries with delayed data (e.g., Curacao; Fig. 3). However, cases estimated by our highly informed model late in the epidemic were pretty close to PAHO reports (i.e., 1.21 x 10^6^ cases predicted by mid-February 2015). Our models allowed us to anticipate CHIKV cases with high confidence in countries with imported cases dominating the reports (e.g., Unites States; Fig. 3). Early models, however, failed to predict case numbers in latter stages of the epidemic in most countries. This effect was particularly evident in countries with inconsistent, heterogeneous, delayed reports (Fig. 3). Models for all countries clearly were improved when more data was added to predictions (Fig. 4). Hence, we suggest that a data-driven method may increase in accuracy when aimed to predict at different stages of the epidemic with forecasts for short periods of time in advance (e.g., one month instead of six months; Fig. 4).

While exploring and assessing vector-borne disease transmission models that might inform us about CHIKV ecology, we noted that true, first-principles transmission models have been developed for a limited suite of vector-borne diseases, particularly malaria and dengue (e.g., [43, 44]), and that models for other vector-borne disease systems have, for the most part, simply been adapted from these base models. Coarse scale predictions based on such transmission models will thus be limited in their applicability to other, more novel, surveillance-limited, large geographic-range, and less-well-studied disease systems such as CHIKV. We were particularly concerned about the effects of parameter selection for these models and their extrapolation to continental extents. Classic disease transmission models are a powerful tool with which to understand epidemics at the population level (e.g., SIR models; [45]), but they require parameters that may be difficult to estimate for a vast diversity of environmental and social scenarios as in the case of the CHIKV epidemic across the Americas (e.g., climate and social features in Canada vs. Colombia). Indeed, traditional transmission models require parameter estimates that may be lacking for the disease, region, species, and scales of interest [46]. Given the limited availability of disease parameters, importing parameters from other studies may provide insights on plausible patterns of the disease ecology, and such imported parameters may (or may not) match with the ecological features of the system where they will be applied [47]. In contrast, we explored simpler, less parameterized approaches. The data-driven approach we used may have implicit the diversity and complexity of the phenomenon at hand. Our approach is most applicable in situations of limited data; however, because we used a data-driven approach, extremely biased or incomplete surveillance and reporting will be able to cause errors and problems, however, the method make such errors identifiable (Fig. 3).

With the Asian CHIKV lineage circulating in the Americas initially in the Caribbean, estimating air traffic is key to understanding CHIKV translocation to uninfected countries [1, 48]. Our measures of city-to-city pairwise airline passenger fluxes were derived and validated based on large data sets and empirical models, and provide good detail on passenger movements, at least at the level of movements of people among countries. Industry data are available and would provide greater detail, but they are apparently extremely costly, and we found them also extremely difficult to access and purchase.

CHIKV reports for most countries started with low numbers of imported cases, followed by dramatic increases once the virus developed autochthonous transmission. These increases of local case numbers often fit an exponential model in early stages of the epidemic, and indeed exponential growth in numbers of cases in the early stage of the CHIKV epidemic in the Americas has been noted previously [5]. Models of focal transmission rates with this form of growth may eventually estimate numbers of infections higher than the total population of a country, which is conceivable in terms of re-infections, but probably just reflects inappropriate model extrapolation. Studies of IgM and IgG antibodies may inform about the acute or convalescent status (or both) of patients, allowing medical professionals to identify individuals with re-infections [49] and estimate true prevalences more accurately.

Numbers of cases may be underrepresented more generally considering asymptomatic individuals [8], under-diagnosis, and lack of reports even of confirmed patients. While some countries provided detailed data for early stages of the epidemic (e.g., Saint Martin, Martinique, Guadeloupe), other countries had limited surveillance effort, with official reports that did not admit the total number of laboratory confirmed cases (e.g., Guatemala; Escobar *pers. obs.*). On the other hand, some areas may be overrepresented as a consequence of incorrect reports based on suspected cases. Antibody test-positive samples from suspicious CHIKV patients may range 29-69 % positivity, illustrating the need for differential diagnosis of, for example, dengue fever [50].

Strikingly, numbers of cases in adjacent areas like Sint Maarten and Saint Martin showed important contrasts in numbers of cases reported (i.e., 470 vs. 5,623 respectively) and prevalences calculated (i.e., 1.26 vs. 16.42 respectively). This pattern may respond to demographic, cultural, and social features of each country. For an artifactual example, whereas the Dominican Republic had 524,381 cases and 5.7 % prevalence, Haiti reported 64,709 cases, for a 0.6 % prevalence, likely associated to differences in availability of diagnostic tests and under-reporting [50]. That is, social factors instead of ecological features driving real transmission appear to be prevailing in these two countries that share the same island.

All countries except the United States showed a pattern of high incidence in early stages followed by a reduction of reports. We found that some countries showed a high number of case reports at the time that other countries in the same region showed an interruption in reporting (e.g., Dominican Republic vs. Colombia). As a consequence, we propose the term “surveillance fatigue” to refer to the reduction of collection, reporting, and publication of epidemiological data after explosive and sustained disease outbreak events*,* resulting in continued increase of transmission and infection, even after the fatigue phase. Surveillance fatigue may also reflect a reduction of assistance of infected people to health care facilities given the simplicity of the disease treatment (e.g., acetaminophen), resulting in an artificial reduction of case numbers after the recognition of the epidemic. Models calibrated with data on early stages may inform better about real incidence of cases in countries showing patterns of surveillance fatigue (Fig. 2 and Additional file 3). Models based on data generated during the surveillance fatigue stage should be considered with caution when developing intervention plans during epidemics, as they will give the impression of damping out of infection rates.

Incorporating imported cases in our predictions allowed us to anticipate CHIKV occurrence in countries with lack of local transmission, *via* air traffic data. For example, cases estimated for the United States were influenced largely by CHIKV prevalence in countries with high passenger flow and consequent importations. Models of local transmission were weak in predicting the fatigue state of the surveillance, based on data from early stages of the epidemic characterized by high transmission rates. Curve shapes resulting from surveillance fatigue can also be the result of seasonal variations of local climate, reducing mosquitoes abundance and activity [51], host immunity mitigating symptoms of re-infections [52], or effective disease control efforts from public health institutions. Previous studies assessing the effectiveness of *Aedes* control strategies had shown low robustness of assessment methodologies, thus, linking the effects of control programmes on disease prevalence is still a challenge [53]. Given the variety of factors that may influence the number of cases reported, ranging from social to climate features, a data-driven method may be a parsimonious approach by which to anticipate case numbers from a diverse epidemiological scenario, with robust predictions when more data are added to the model and short time periods are predicted in advance (Fig. 5). Whether our transmission model performs better than other classic approaches is a question that should be explored statistically using data under controlled experimental conditions. Accurate data from an epidemic among different countries may be hard to derive, so such studies may fall in the field of virtual ecology, where the real number of disease cases and levels of surveillance bias is well known [30, 54]. The application of virtual ecology in epidemiology to compare transmission models is an area that deserves special attention and has a promising future [54], given that it may help to elucidate the best model algorithms and approaches for forecast disease spread.

### Transmission hotspots

**Fig. 6:**
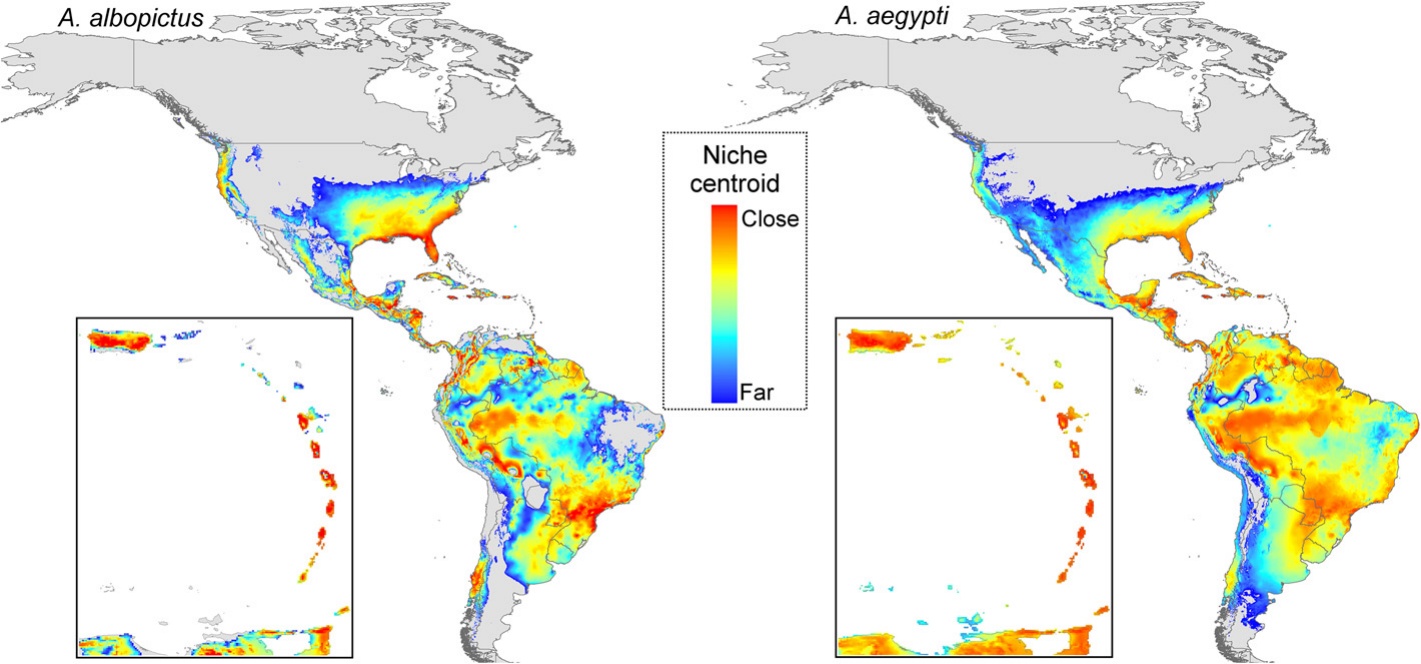
Hotspots of Chikungunya transmission risk, as measured in terms of distance to the niche centroid. Red areas are those with environments close the niche centroid, denoting areas with high potential vector abundance. Caribbean countries are shown in the inset

Suitable areas for occurrence of the two mosquito species were found across all countries in the Americas. However, areas of high suitability, in terms of distance to the niche centroid, were concentrated in tropical and subtropical latitudes. Indeed, areas considerably suitable for *A. aegypti* matched with countries of initial reports of CHIKV cases in the Caribbean (Fig. 6), suggesting that the introduction of the virus into the Americas was to “fertile soil” in terms of holding highly competent vector populations. We identified and proposed hotspot areas of transmission risk based on niche centroid distances (Fig. 6). We found that *A. aegypti* may find more ideal areas to sustain high transmission rates, particularly in Haiti, Dominican Republic, Puerto Rico, Guadeloupe, Dominica, Martinique, St Lucia, Saint Vincent and the Grenadines, and Grenada, plus on the mainland in coastal Venezuela and Brazil, across Central America, and in the lowlands of Peru and Bolivia. *Aedes albopictus*, on the other hand, has areas of high transmission potential in the southeastern United States, southern Brazil, central Chile, Central America, and across the Andes Mountains in Bolivia. Countries closest to the niche centroid had higher CHIKV prevalences (*y* = 0.0004747*log(*x*); *P* = 0.002), but we found no significant association between GDP values and reported prevalences by country (*r*^2^ = 0.002; df = 7; *P* = 0.085).

Our macroecological and biogeographic consideration, instead of classic correlative approaches, allowed us to appreciate the global biogeographic potential of CHIKV transmission compared to regional models of suitability [55]. The niche centroid idea is not novel and it has been proposed theoretically [20] and tested empirically in ecology [22, 28]; it offers a linkage between geographic range and population biology of species [21, 22]. Our novel application of the niche-centrality paradigm for an infectious disease at coarse geographic extents (Fig. 6), may promote the use of this technique to assess abundance patterns and genetic structure in infectious disease systems to inform mitigation strategies [21, 22]. At local scales, CHIKV *R*_0_ is expected to range between less than unity and greater than 8 for middle and low latitudes, respectively, with highest values expected at the environmental centroid [27]. Our niche estimation was based on fine scale climate data from spatial interpolations [37], however, we advise caution when comparing these findings on virus potential transmission from our vectors’ suitability maps, due to the global scale and environmental data employed during the ENMs calibration. Comparisons between different spatial scales may fail to show an agreement in results [56]. The lack of agreement in two models developed at different scales is not novel in ecology, and has been termed the Beale fallacy (dissimilar patterns resulting from incorrect comparisons of models developed at different scales; [57]).

ENM of *A. albopictus* and *A. aegypti* have been developed previously under different approaches [4, 55, 58–62]. For instance, ENM are commonly calibrated at regional scale, showing high suitability in the sampled areas as a result of the correlative nature of the algorithms employed [46, 55, 58, 63], calibrating a species’ ENM based on fewer occurrences may result in incomplete distributional estimations [64]. Current ENM for *A. albopictus* and *A. aegypti*, developed at global scales to anticipate their distribution under future climate conditions, suggest that both species may find suitable conditions in different, currently-unsuitable areas, given their ecological plasticity and their impressive dispersal abilities [4]. Campbell et al. [4], developed global ENMs of CHIKV vectors and mitigate sampling bias *via* a sampling bias background and generating binary outputs. ENMs based on the entire species distribution and estimating the niche centroid to find areas close or far to such centrality (i.e., areas more or less suitable) could reduce sampling bias effects, and may provide biological meaning to the continuous surfaces generated by the model, as has been shown in empirical experiments [21, 22, 28, 29]. Here, our ENMs based on niche centroid distance showed that suitability patterns across the Americas agreed with prevalences of CHIKV.

## Conclusions

The CHIKV transmission model and transmission-hotspot maps presented here are methodologically valuable, as we generated predictions based exclusively on open-access tools and data. However, this approach has some important limitations. First, our transmission model is data-driven, so, poor-quality data can generate poor predictions. We found that continuous reporting by countries improved model predictions, whereas interrupted and delayed epidemiological reports generated poor forecasts, as exemplified by the intermittent and fatigued surveillance and reporting pattern of Curacao (Fig. 3), El Salvador, and Haiti (Additional file 3). Since scientific literature regarding CHIKV occurrence in the Americas will inevitably be published with 2–6 months’ delay [65], official reports play a key role in early notification of epidemiological shifts [66] and in enabling predictive modeling. The PAHO online interface could improve the collection and storage of epidemiological records to facilitate early use of data and fast generation of results to inform interventions, reducing the time between data collection and analyses.

Second, we explored city-to-city, hemisphere-wide passenger flow through an average estimation (Additional file 1). Important seasonal differences clearly exist for air traffic through the year, but were not included in our implementation. This issue can potentially be addressed using our data source *via* incorporation of seasonal trend information regarding movement pulses such as home visits by migrant workers, tourism windows, and holiday schedules, among others. Clearly, the relative simplicity of our air travel data represents a limitation of the approach; however, given that this dataset was a good proxy to estimate imported cases for some countries (e.g., United States), we release the estimated air traffic data for further testing, including the origin and destination of passengers (Additional file 4).

Third, we modeled virus translocation *via* air travel only, neglecting surface transport in the form of ground and sea travel, which are also potentially important in terms of movements of infected vectors or passengers [67]. However, considering the importance of air travel in modern society, the fast movement between countries, the massive passenger flow, and the recent nature of the CHIKV invasion of the Americas, we expected the air travel data to capture representative patterns of movement most relevant to the virus’ spread in the Americas, at least at these initial stages. An alternative to our travel-based approach would be by compiling national immigration data from entry ports, but these data may not be available in most countries in Latin America.

Fourth, an easy improvement to our model selection approach for local transmission would be to use the Akaike information criterion (AIC) to choose among alternative models, which would be simple to implement and automate. It would provide an indication of which population growth algorithm should be used, with decisions based on goodness-of-fit of each model to each country’s accumulation of autochthonous cases. In this particular exploration, however, we decided not to use such metrics, to allow careful identification and custom consideration of biases and other non-biological factors discussed above that affect numbers of cases reported in ways that have nothing (or little) to do with local transmission rates.

Predictions at early stages of the epidemic had high uncertainty when compared with more informed models (Fig. 4), which should be considered in control strategies based on predictions at early stages of epidemics. Our predictions fit fairly consistently with posterior reports (e.g., Fig. 5), inspiring some confidence in our model outputs. We emphasize the perhaps-dominant role that reporting biases can play in the PAHO case-occurrence data sets. These biases mean that biological factors may at times take a back seat to human-driven factors. The outcomes of our work consist of detailed maps and tables of probabilities and likely numbers of cases on a pixel-by-pixel, or region-by-region basis. These products can feed directly into real-world mitigation strategies *via* identification of areas most at risk of arrival of new cases *via* importation. The advantage of such information for CHIKV, as compared (say) to Ebola virus cases, is that arriving cases, if aware of their CHIKV-positive status, simply need to avoid exposure to mosquitoes carefully, and no further transmission should occur.

## Additional files

Additional file 1: Methods to estimate the passenger flow across countries. (DOCX 2382 kb) Additional file 2: Theoretical description of the ecological niche model for mapping multivariate centroid of suitable conditions. (DOCX 63 kb) Additional file 3: Countries in surveillance ‘fatigue’ stage. (DOCX 175 kb) Additional file 4: Air traffic data used to estimate passenger flow among countries in the Americas. The table includes direct flights between countries with no stops, flights with one or two stops for flight connections, and a summary of total estimated number of passengers between countries in the Americas. (CSV 108 kb)

## Abbreviations

%: percentage
AIC: akaike information criterion
CHIKV: Chikungunya virus
ENM: ecological niche models/ecological niche modeling
GDP: gross domestic product
M: million
MVE: minimum-volume ellipsoid
NI: number of cases imported by week
NL: number of cases generated in a country by local transmission by week
NT: cumulative number of individuals infected by week
PAHO: Pan American Health Organization
PCA: principal component analysis
S: supplementary material
U.S: United States

## References

[1] Leparc-Goffart I, Nougairede A, Cassadou S, Prat C, De Lamballerie X (2014). Chikungunya in the Americas. Lancet.

[2] Cassadou S, Boucau S, Petit-Sinturel M, Huc P, Leparc-Goffart I, Ledrans M (2014). Emergence of chikungunya fever on the French side of Saint Martin island, October to December 2013. Eurosurveillance.

[3] Vega-Rúa A, Zouache K, Girod R, Failloux A-B, Lourenço-de-Oliveira R (2014). High level of vector competence of *Aedes aegypti* and *Aedes albopictus* from ten American countries as a crucial factor in the spread of chikungunya virus. J Virol.

[4] Campbell LP, Luther C, Moo-Llanes D, Ramsey JM, Danis-Lozano R, Peterson AT. Climate change influences on global distributions of dengue and chikungunya virus vectors. Philos Trans R Soc B Biol Sci. 2015;370:20140135–5. 10.1098/rstb.2014.0135 PMC4342968 25688023

[5] Cauchemez S, Ledrans M, Poletto C, Quenel P, de Valk H, Colizza V, Boëlle PY. Local and regional spread of chikungunya fever in the Americas. Eurosurveillance. 2014;19:1–9. 10.2807/1560-7917.es2014.19.28.20854 PMC4340072 25060573

[6] McCarthy KP, Fletcher RJ, Rota CT, Hutto RL (2012). Predicting species distributions from samples collected along roadsides. Conserv Biol.

[7] Kadmon R, Farber O, Danin A (2004). Effect of roadside bias on the accuracy of predictive maps produced by bioclimatic models. Ecol Appl.

[8] Sissoko D, Moendandze A, Malvy D, Giry C, Ezzedine K, Solet JL, Pierre V. Seroprevalence and risk factors of chikungunya virus infection in Mayotte, Indian Ocean, 2005–2006: A population-based survey. PLoS ONE. 2008;3:e3066. 10.1371/journal.pone.0003066 PMC2518850 18725980

[9] Pialoux G, Gaüzère BA, Jauréguiberry S, Strobel M (2007). Chikungunya, an epidemic arbovirosis. Lancet Infect Dis.

[10] Johansson MA, Powers AM, Pesik N, Cohen NJ, Staples JE (2014). Nowcasting the spread of chikungunya virus in the Americas. PLoS ONE.

[11] Pan American Health Organization. Chikungunya: Statistic Data. 2015. [Online]. Available: http://bit.ly/1zgnYGE. [accessed: 17-Feb-2015]

[12] Brockmann D, Helbing D (2013). The hidden geometry of complex, network-driven contagion phenomena. Science.

[13] Official Airline Guide. Traffic Analyser.OAG, 2015. [Online]. Available: www.oag.com. [Accessed: 07-Nov-2014].

[14] Megginson D. OurAirports. Runways, 2015. [Online]. Available: www.ourairports.org. [Accessed: 07-Nov-2014].

[15] Wikipedia Foundation. Wikipedia, The Free Encyclopedia. 2004. [Online]. Available: http://en.wikipedia.org/. [Accessed: 01-Jan-2015].

[16] U.S. Department of Transportation: Bureau of Trasportation Statistics. Office of the Assistant Secretary for Research and Technology, 2014. [Online]. Available: www.transtats.bts.gov. [Accessed: 07-Nov-2014].

[17] Wikipedia Foundation. World’s busiest passenger air routes..2014. [Online]. Available: en.wikipedia.org/wiki/World’s_busiest_passenger_air_routes. [Accessed: 07-Nov-2014].

[18] Wikipedia Foundation. List of countries and dependencies by population. 2014. [Online]. Available: https://en.wikipedia.org/wiki/List_of_countries_and_dependencies_by_population. [Accessed: 07-Nov-2014].

[19] Peterson AT, Soberón J, Pearson RG, Anderson RP, Martínez-Meyer E, Nakamura M, et al. Ecological Niches and Geographic Distributions. New Jersey: Princeton University Press; 2011.

[20] Holt RD (2009). Bringing the Hutchinsonian niche into the 21st century: Ecological and evolutionary perspectives. Proc Natl Acad Sci USA.

[21] Yañez-Arenas C, Peterson AT, Mokondoko P, Rojas-Soto O, Martínez-Meyer E (2014). The use of ecological niche modeling to infer potential risk areas of snakebite in the Mexican state of Veracruz. PLoS ONE.

[22] Martínez-Meyer E, Diaz-Porras D, Peterson AT, Yañez-Arenas C. Ecological niche structure and rangewide abundance patterns of species. Biol Lett. 2012;9:20120637–7. 10.1098/rsbl.2012.0637 PMC3565484 23134784

[23] Maguire BJ (1973). Niche response structure and the analytical potential of its relationships to the habitat. Am Nat.

[24] Drake JM (2015). Range bagging: A new method for ecological niche modelling from presence-only data. J R Soc Interface.

[25] Soberón J, Nakamura M (2009). Niches and distributional areas: Concepts, methods, and assumptions. Proc Natl Acad Sci USA.

[26] Ruiz-Moreno D, Vargas IS, Olson KE, Harrington LC (2012). Modeling dynamic introduction of chikungunya virus in the United States. PLoS Negl Trop Dis.

[27] Perkins TA, Metcalf CJE, Grenfell BT, Tatem AJ (2015). Estimating drivers of autochthonous transmission of chikungunya virus in its invasion of the Americas. PLOS Curr Outbreaks.

[28] Manthey JD, Campbell LP, Saupe EE, Soberón J, Hensz CM, Myers CE, Owens HL, Ingenloff K, Peterson A, Barve N, Lira-Noriega A, Barve V. A test of niche centrality as a determinant of population trends and conservation status in threatened and endangered North American birds. Endanger Species Res. 2015;26:201–8.

[29] Lira-Noriega A, Manthey JD (2014). Relationship of genetic diversity and niche centrality: A survey analysis. Evolution.

[30] Qiao H, Soberón J, Peterson AT (2015). No silver bullets in correlative ecological niche modelling: insights from testing among many potential algorithms for niche estimation. Methods Ecol Evol.

[31] Qiao H, Escobar LE, Soberón J, Campbell L, Peterson AT. NicheA. Version 3.0.1 2015. [Online]. Available: http://nichea.sourceforge.net/. [Accessed: 12-Jan-2015].

[32] Peterson AT (2014). Mapping Disease Transmission Risk: Enriching Models Using Biology and Ecology.

[33] National Museum of Natural History. VectorMap Data Portal. Smithsonian Institution, 2014. [Online]. Available: http://vectormap.org/. [Accessed: 05-May-2014].

[34] Belbin L. Atlas of Living Australia. 2014. [Online]. Available: http://www.ala.org.au/. [Accessed: 05-May-2014].

[35] Centro de Referência em Informação Ambiental: speciesLink. 2014. [Online]. Available: http://splink.cria.org.br/. [Accessed: 05-May-2014].

[36] Global Biodiversity Information Facility: GBIF Species Database. 2014. [Online]. Available: http://www.gbif.org/. [Accessed: 05-May-2014].

[37] Hijmans RJ, Cameron SE, Parra JL, Jones PG, Jarvis A (2005). Very high resolution interpolated climate surfaces for global land areas. Int J Climatol.

[38] Van AS, Rousseeuw P (2009). Minimum volume ellipsoid. Wiley Interdiscip Rev Comput Stat.

[39] Qiao H, Peterson AT, Campbell LP, Soberón J, Ji L, Escobar LE. NicheA: Creating virtual species and ecological niches in multivariate environmental scenarios. Ecography 2015:In press.

[40] R Core Team (2015). R: A Language and Environment for Statistical Computing.

[41] ESRI (2015). ArcGIS Desktop: Release 10.2.

[42] Walton DA, Ivers LC (2011). Responding to cholera in post-earthquake Haiti. N Engl J Med.

[43] Esteva L, Vargas C (1998). Analysis of a dengue disease transmission model. Math Biosci.

[44] Craig M, Le Sueur D, Snow B (1999). A climate-based distribution model of malaria transmission in sub-Saharan Africa. Parasitol Today.

[45] Keeling MJ, Rohani P (2007). Modeling Infectious Diseases in Humans and Animals.

[46] Kearney M, Porter WP, Williams C, Ritchie S, Hoffmann AA (2009). Integrating biophysical models and evolutionary theory to predict climatic impacts on species’ ranges: The dengue mosquito *Aedes aegypti* in Australia. Funct Ecol.

[47] Pech RP, Hone J (1988). A model of the dynamics and control of an outbreak of foot and mouth disease in feral pigs in Australia. J Appl Ecol.

[48] Khan K, Bogoch I, Brownstein JS, Miniota J, Nicolucci A, Hu W, Nsoesie EO, Cetron M, Creatore MI, German M, Wilder-Smith A. Assessing the origin of and potential for international spread of chikungunya virus from the Caribbean. PLoS Curr. 2014;6:1–11. 10.1371/currents.outbreaks.2134a0a7bf37fd8d388181539fea2da5 PMC4055609 24944846

[49] Staples JE, Breiman RF, Powers AM (2009). Chikungunya fever: An epidemiological review of a re-emerging infectious disease. Clin Infect Dis.

[50] Van Bortel W, Dorleans F, Rosine J, Blateau A, Rousseau D, Matheus S, Leparc-Goffart I, Flusin O, Prat CM, Césaire R, Najioullah F, Ardillon V, Balleydier E, Carvalho L, Lemaître A, Noël H, Servas V, Six C, Zurbaran M, Léon L, Guinard A, van den Kerkhof J, Henry M, Fanoy E, Braks M, Reimerink J, Swaan C, Georges R, Brooks L, Freedman J,. Chikungunya outbreak in the Caribbean region, December 2013 to March 2014, and the significance for Europe. Eurosurveillance. 2014;19:20759. 10.2807/1560-7917.es2014.19.13.20759 24721539

[51] Stewart-Ibarra AM, Lowe R (2013). Climate and non-climate drivers of dengue epidemics in southern coastal Ecuador. Am J Trop Med Hyg.

[52] Her Z, Malleret B, Chan M, Ong EKS, Wong SC, Kwek DJC, Tolou H, Lin RTP, Tambyah PA, Renia L, Ng LFP. Active infection of human blood monocytes by chikungunya virus triggers an innate immune response. J Immunol. 2010;184:5903–13. 10.4049/jimmunol.0904181 20404274

[53] Heintze C, Garrido MV, Kroeger A (2007). What do community-based dengue control programmes achieve? A systematic review of published evaluations. Trans R Soc Trop Med Hyg.

[54] Leroy B, Meynard CN, Bellard C, Courchamp F. virtualspecies, an R package to generate virtual species distributions. Ecography 2015, in press:1–9.

[55] Fischer D, Thomas SM, Neteler M, Tjaden NB, Beierkuhnlein C (2014). Climatic suitability of *Aedes albopictus* in Europe referring to climate change projections: Comparison of mechanistic and correlative niche modelling approaches. Eurosurveillance.

[56] Astorga F, Poo-Muñoz DA, Escobar LE, Medina-Vogel G (2015). In response to: “Increased dog population and potential for bat-borne rabies spillover in Chile in response to ‘Dog management, abundance and potential for bat-borne rabies spillover in Chile’ by Astorga et al. [Prev. Vet. Med. 118:397–405]” by Acosta-Jam. Prev Vet Med.

[57] Escobar LE, Peterson AT, Favi M, Yung V, Pons DJ, Medina-Vogel G (2013). Ecology and geography of transmission of two bat-borne rabies lineages in Chile. PLoS Negl Trop Dis.

[58] Machado-Machado EA (2012). Empirical mapping of suitability to dengue fever in Mexico using species distribution modeling. Appl Geogr.

[59] Neteler M, Metz M, Rocchini D, Rizzoli A, Flacio E, Engeler L, Guidi V, Lüthy P, Tonolla M. Is Switzerland suitable for the invasion ofAedes albopictus? PLoS ONE. 2013;8:e82090. 10.1371/journal.pone.0082090 PMC3862574 24349190

[60] Bhatt S, Gething PW, Brady OJ, Messina JP, Farlow AW, Moyes CL, Drake JM, Brownstein JS, Hoen AG, Sankoh O, Myers MF, George DB, Jaenisch T, Wint GRW, Simmons CP, Scott TW, Farrar JJ, Hay SI. The global distribution and burden of dengue. Nature. 2013;496:504–7. 10.1038/nature12060 PMC3651993 23563266

[61] Arboleda S, Jaramillo-O N, Peterson AT (2012). Spatial and temporal dynamics of *Aedes aegypti* larval sites in Bello, Colombia. J Vector Ecol.

[62] Medley KA (2010). Niche shifts during the global invasion of the Asian tiger mosquito, *Aedes albopictus* Skuse (Culicidae), revealed by reciprocal distribution models. Glob Ecol Biogeogr.

[63] Miller MJ, Loaiza JR (2015). Geographic expansion of the invasive mosquito *Aedes albopictus* across Panama - Implications for control of dengue and chikungunya viruses. PLoS Negl Trop Dis.

[64] Escobar LE, Lira-Noriega A, Medina-Vogel G, Peterson AT (2014). Potential for spread of White-nose fungus (*Pseudogymnoascus destructans*) in the Americas: Using Maxent and NicheA to assure strict model transference. Geospat Health.

[65] Diaz Y, Carrera JP, Cerezo L, Arauz D, Guerra I, Cisneros J, Armien B, Botello AM, Arauz AB, Gonzalez V, Lopez Y, Moreno L, Lopez-Verges S, Moreno BA. Chikungunya virus infection: First detection of imported and autochthonous cases in Panama. Am J Trop Med Hyg. 2015;92:482–5. 10.4269/ajtmh.14-0404 PMC4350534 25601996

[66] Frieden TR (2013). Government’s role in protecting health and safety. N Engl J Med.

[67] Woolley-Meza O, Thiemann C, Grady D, Lee JJ, Seebens H, Blasius B, Brockmann D. Complexity in human transportation networks: A comparative analysis of worldwide air transportation and global cargo-ship movements. Eur Phys J B. 2011;84:589–600.

[68] Brownstein JS, Freifeld CC, Reis BY, Mandl KD (2008). Surveillance Sans Frontières: Internet-based emerging infectious disease intelligence and the HealthMap project. PLoS Med.

