# Supplementary material for: Forecasting Chikungunya spread in the Americas via data-driven empirical approaches
Source: Parasit Vectors. 2016 Feb 29;9:112. doi: 10.1186/s13071-016-1403-y (PMC4772319; doi:10.1186/s13071-016-1403-y)
Supplement: Additional file 1: — Methods to estimate the passenger flow across countries. (DOCX 2382 kb) [file 13071_2016_1403_MOESM1_ESM.docx]

**Supplementary material S1. Methods to estimate the passenger flow across countries.**

**
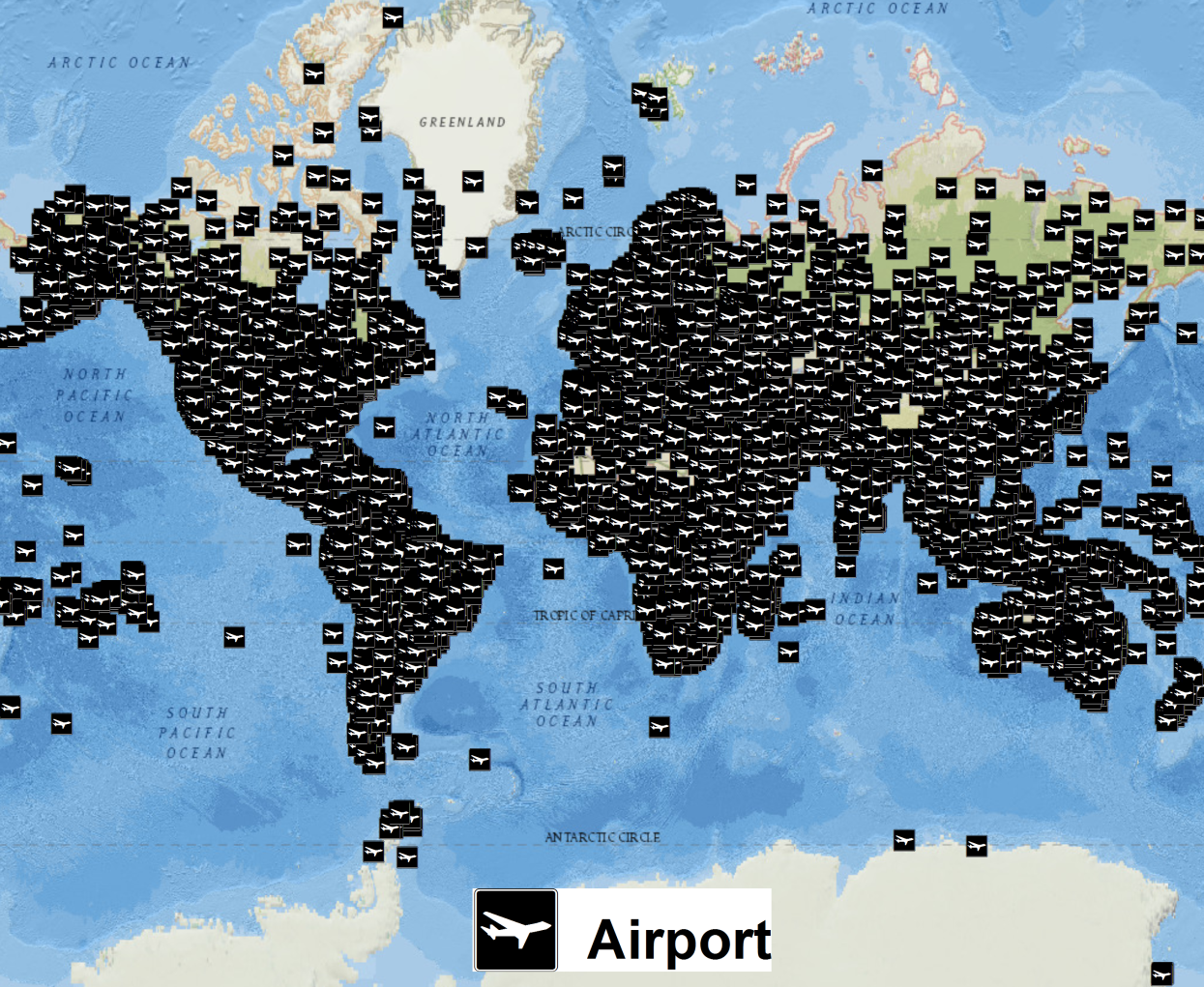
**

**Figure S1.1. Geographic distribution of airports used to estimate passenger flow.**

Once aircraft routes and passenger data were collected, collated, and formatted (Fig. S1.1), we developed a random forests model to associate route, airport, region, and runway data with passenger flow in the United States-connected flight dataset. The best final model omitted month as a predictor variable, and explained 90.1% of total overall variance in the data set (Fig. S1.2a).

**
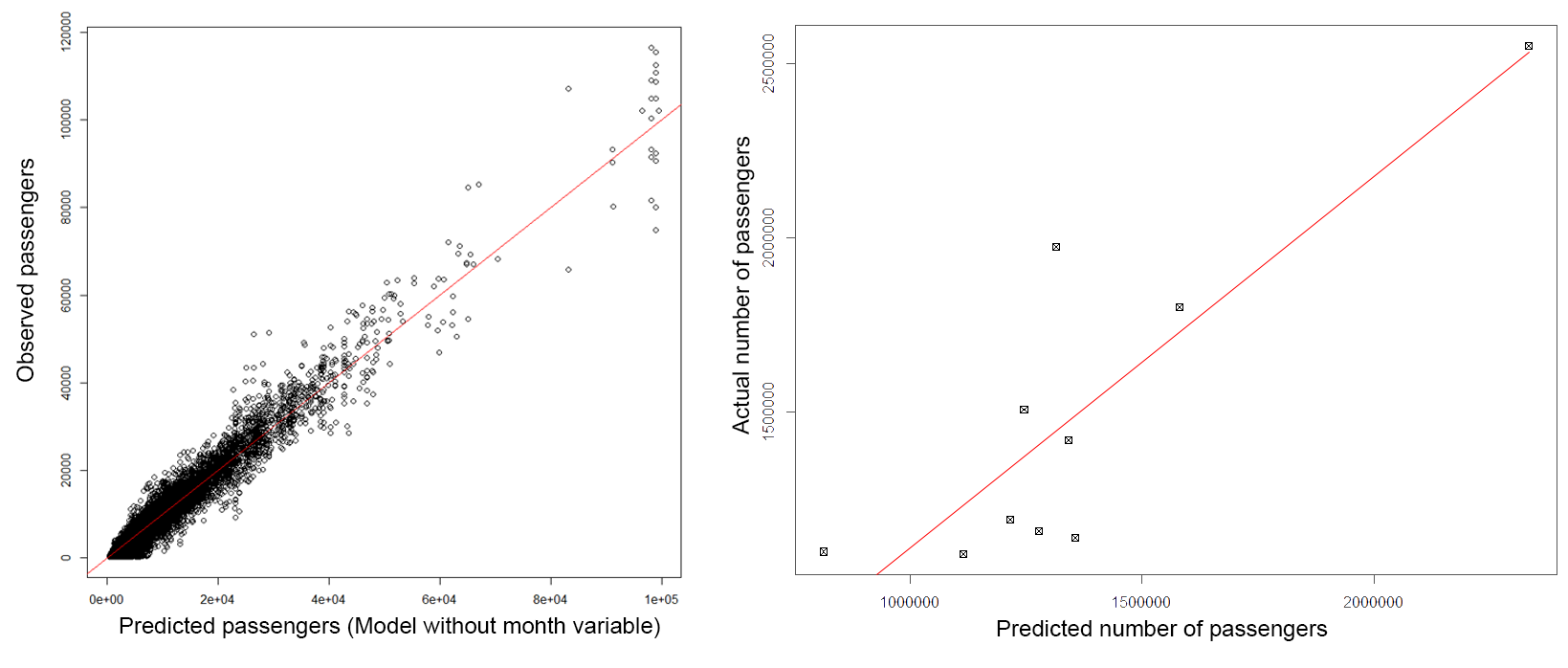
**

**Figure S1.2. Passenger flow model between cities.** (A) model calibration, and (B) model evaluation via independent observed passenger flow.

We validated this correlative model using an independent data set (i.e., the top 10 routes in the world, sourced from Wikipedia^[[1]](#footnote-1)^; Fig. S1.2b): the model explained 73.0% of variation in passenger numbers (*P* = 0.0016, *r*^2^=0.73), indicating considerable predictive power as regards passenger flow. We note that such correlative modeling of passenger flow represents a zero-cost, open-source segment of our methodology that could nonetheless be replaced by industry data, if the high cost were to be outbalanced by desire for less overall variance in the data.

We applied the best random-forests model to all city-city pairs across the Americas to estimate numbers of passengers moving yearly between each pair of points (Fig. S1.3). Thus, based on the passenger flow model (Fig. S1.2), we estimated the city-to-city and country-to-country passenger flow. In the simple diagram of Figure S1.3, arrows R1 to R7 are flight routes between airports. In this example, passengers from city A1 and B1 and from city A2 to B2 were estimated. However, we estimated passenger flow based on all flight route combinations available. The city-pair passengers flow is the sum of the airport-paired passenger flows between two cities (e.g., equations in the bottom left). The final country-to-country passenger flow is the sum of passengers from all routes between these two countries (e.g., equation in the bottom right).

**
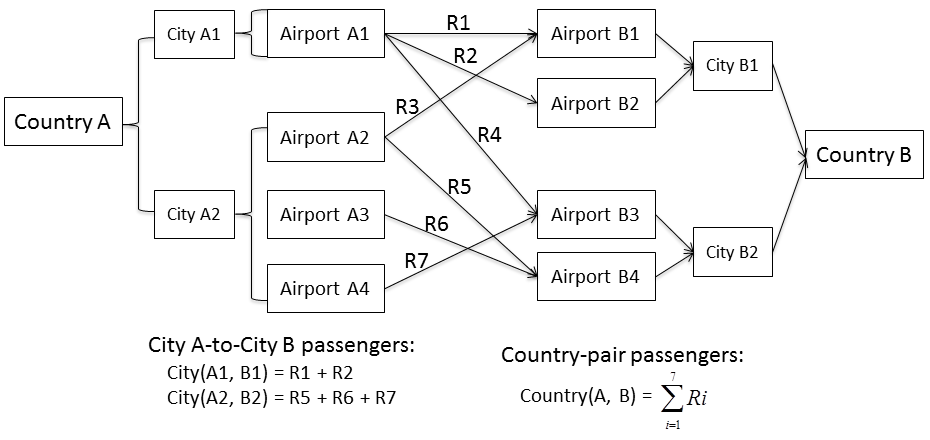
**

**Figure S1.3. Passenger flow model between countries.**

The disadvantages of this approach to assembling a passenger-flow data set are that it cannot manage lost flights (i.e., empty seats) or the full complexity of flight connections that passengers make, even when we reconstructed secondary and tertiary movement of passengers among countries based on assumptions and data available, although the outputs clearly captured degrees of connectivity and lack of connectivity of different airports in different countries (Fig. S1.4). The advantage of this approach, however, was clearly its open availability and zero cost and its transparent derivation from observed data.


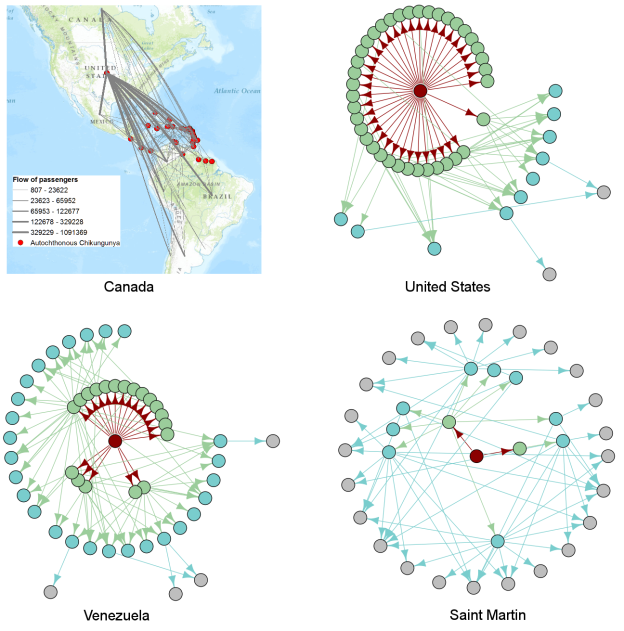


**Figure S1.4. Passenger flow configurations in flights to and from example Latin American and Caribbean countries.** Top left: map showing passenger flow from Canada to Latin American and Caribbean countries, indicating nonstop flights (red circle) and high (thick gray line) or low (fine gray line) amounts of passengers on flights with connections. Network configurations within the hemisphere for United States, Venezuela, and Saint Martin indicating the origin of passengers (red circle), one connection (green circle), second connection (blue circle), and a third connection flights for the final destination (gray circle). Note the sharp contrasts among the countries in degree of connectedness to other countries.

1. World's busiest passenger air routes: en.wikipedia.org/wiki/World's_busiest_passenger_air_routes [↑](#footnote-ref-1)
