# Supplementary material for: Forecasting Chikungunya spread in the Americas via data-driven empirical approaches
Source: Parasit Vectors. 2016 Feb 29;9:112. doi: 10.1186/s13071-016-1403-y (PMC4772319; doi:10.1186/s13071-016-1403-y)

**Supplementary Material S2. Theoretical description of the ecological niche model for mapping distances to multivariate centroids of ecological niches.** (A) Occurrence points (red) are displayed in the environmental space according to climate values available worldwide (gray points). An ecological niche model is estimated in the form of a minimum-volume ellipsoid around occurrences (solid line). (B) The ellipsoid model is categorized in terms of the distance to the ellipsoid centroid (black point). The model is then projected into geographic space, with areas close to the niche centroid as areas of high environmental suitability, while areas at the edge of the niche (i.e., far from the niche centroid) are denoted as areas of low suitability. Environmental conditions outside of the ellipsoid are denoted as unsuitable for the species’ long-term survival. For a good proxy of the fundamental niche, allowing in turn a good estimation of the niche’s centroid, it is critical to utilize occurrence data across the entire geographic distribution of the species.


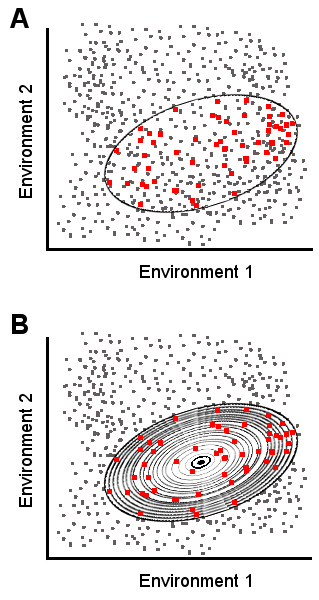

Supplement: Additional file 2: — Theoretical description of the ecological niche model for mapping multivariate centroid of suitable conditions. (DOCX 63 kb) [file 13071_2016_1403_MOESM2_ESM.docx]
