# Supplementary material for: Forecasting Chikungunya spread in the Americas via data-driven empirical approaches
Source: Parasit Vectors. 2016 Feb 29;9:112. doi: 10.1186/s13071-016-1403-y (PMC4772319; doi:10.1186/s13071-016-1403-y)
Supplement: Additional file 3: — Countries in surveillance ‘fatigue’ stage. (DOCX 175 kb) [file 13071_2016_1403_MOESM3_ESM.docx]

**Supplementary material S3. Countries in surveillance ‘fatigue’ stage.**


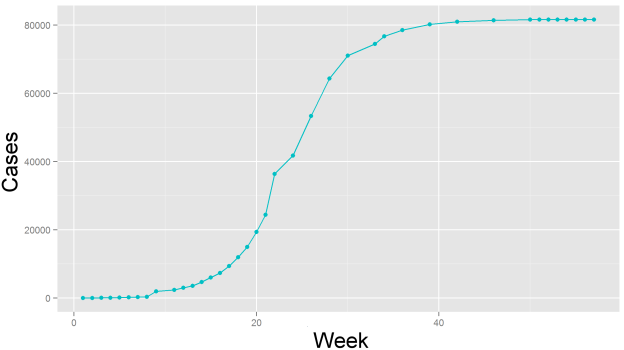


**Figure S3.1. Accumulated Chikungunya cases in Guadalupe.** Local transmission (blue line) was observed since the first reports of chikungunya in the country. Notice that after a raise on the number of cases with exponential growth, reports decrease gradually until they cease, falling into a stage of epidemiological surveillance ‘fatigue.’


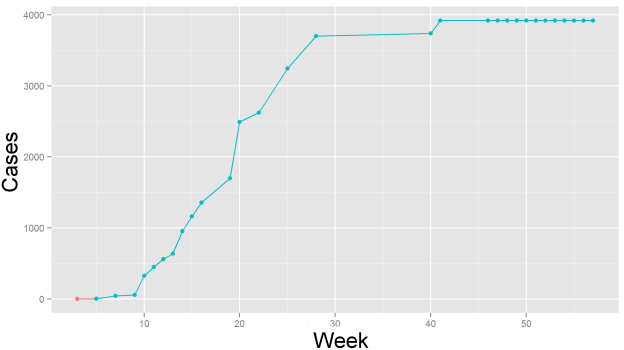


**Figure S3.2. Accumulated Chikungunya cases in Dominica.** Imported cases (red line) and local transmission (blue line) was observed since first month of reports. Dominica experienced a dramatic (linear) increase on the number of cases after autochthonous transmission was established in the country. However, active reporting of cases was observed until week 28; by week 40, reports decreased abruptly and reached zero by week 46.

**
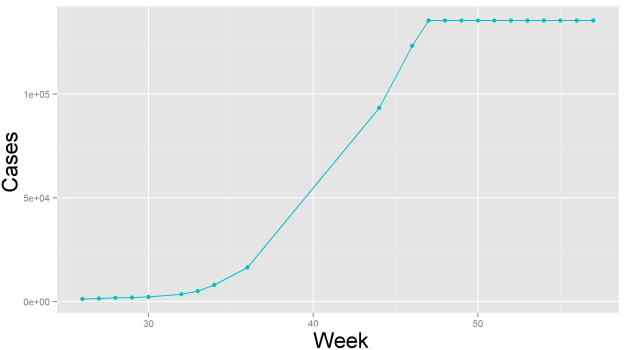
**

**Figure S3.3. Accumulated Chikungunya cases in El Salvador.** El Salvador is a clear example of a country in epidemiological surveillance ‘fatigue’ stage. After exponential increase of cases initially, reports stopped abruptly after 21 weeks.

**
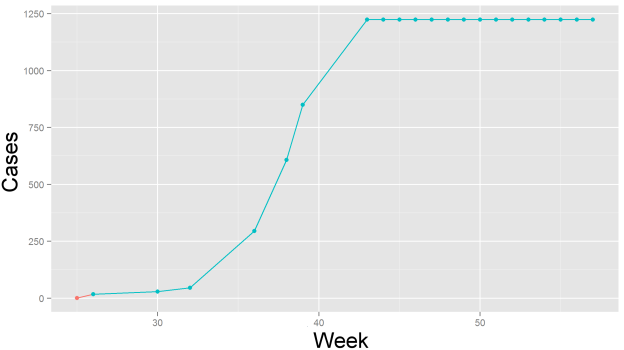
**

**Figure S3.4. Accumulated Chikungunya cases in Suriname.** Chikungunya cases in Suriname increased dramatically after local transmission (blue line) was established in the country. While the pattern of cases should increase following week 40, epidemiological reports ceased by week 43.

**
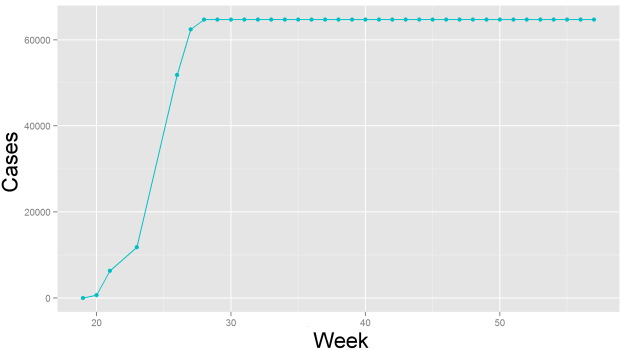
**

**Figure S3.5. Accumulated Chikungunya cases in Haiti.** After accumulating 64,695 cases of chikungunya during 9 weeks of surveillance, Haiti stopped reporting by week 28.
